# Conventional Cardiopulmonary Resuscitation Versus Extracorporeal Membrane Oxygenation-Assisted CPR in Children: A Retrospective Analysis of Outcomes and Factors Associated with Conversion from the Former to the Latter

**DOI:** 10.3390/children12030378

**Published:** 2025-03-18

**Authors:** Adrian C. Mattke, Eugene Slaughter, Kerry Johnson, Michelle Low, Kim Betts, Kristen S. Gibbons, Renate Le Marsney, Supreet Marathe

**Affiliations:** 1 Department of Paediatric Intensive Care, Queensland Children’s Hospital, Brisbane 4101, Australia; kerry.johnson3@health.qld.gov.au; 2 Child Health Research Centre, The University of Queensland, Brisbane 4102, Australia; e.slaughter@uq.edu.au (E.S.); k.gibbons@uq.edu.au (K.S.G.); r.lemarsney@uq.edu.au (R.L.M.); 3 School of Medicine, The University of Queensland, Brisbane 4006, Australia; supreet.marathe@health.qld.gov.au; 4 Department for Cardiothoracic Surgery, Queensland Children’s Hospital, Brisbane 4101, Australia; 5 Department of Paediatrics, University Malaya Medical Centre, Kuala Lumpur 59100, Malaysia; mlsyee@ummc.edu.my; 6 School of Population Health, Curtin University, Perth 6102, Australia; kim.betts@curtin.edu.au

**Keywords:** child, cardiopulmonary resuscitation, extracorporeal membrane oxygenation, survival

## Abstract

Background/Objectives: Conventional cardiopulmonary resuscitation (CCPR) has been the foundational resuscitation approach for decades. Where CCPR is unsuccessful, extracorporeal membrane oxygenation-assisted CPR (ECPR) may improve outcomes. Predicting failure of CCPR and immediate need for ECPR is difficult, and data are lacking. In this retrospective analysis, we analysed both factors that are associated with conversion from CCPR to ECPR and survival outcomes for each event. Methods: Patients having a CPR event that occurred in the PICU between 2016 and 2022 were included. Pre-CPR-event clinical and laboratory data were collected. We recorded whether CPR was converted to ECPR and documented patient outcomes. Results: 201 CPR events occurred in 164 children, with 45 events converted from CCPR to ECPR. Time to ROSC or time to ECMO flow was (median [IQR]) 2 (1.5) min for CCPR events and 37 (21.60) min for ECPR events. The maximum pre-CPR-event lactate values were 1.8 mmol/L for CCPR and 4.5 mmol/L for ECPR events. Respiratory arrest preceded 35.3% of CCPR and 4.4% of ECPR events. PICU mortality was 27.8% for CCPR and 50% for ECPR events. Most deaths occurred because of withdrawal of life-sustaining treatments. In a multivariable analysis, cardiac surgical diagnosis, pre-CPR-event lactate, as well as duration of CPR were associated with conversion from CCPR to ECPR. Conclusions: Our study demonstrates that pre-CPR-event lactate concentrations and duration of arrest should alert clinicians to a high likelihood of needing ECPR, while a preceding respiratory arrest may indicate a low likelihood. Mortality post CCPR is significant, mainly due to overall illness severity rather than the consequences of the CPR event.

## 1. Introduction

Conventional cardiopulmonary resuscitation (CCPR) has been the foundational approach to resuscitating children and adults for decades, providing a lifeline through manual chest compressions and ventilatory support [1]. CCPR was codified in guidelines for adults and children as early as 1979 and 1997, respectively [2,3]. However, its effectiveness is often limited by various factors, including the quality of compressions and the patient’s underlying health conditions, leading to a survival rate from in-hospital cardiac arrest of only around 50% [4,5]. The advent of extracorporeal membrane oxygenation (ECMO)-assisted CPR (ECPR) offers a potentially superior alternative, promising to redefine the boundaries of life-saving care [6]. In fact, ECPR has demonstrated superior survival rates when compared to CCPR, particularly in cases with longer duration of CPR efforts, and has also been associated with positive effects on neurocognitive outcomes post arrest, reported to be as high as 60% [7,8,9,10]. Current guidelines suggest converting from CCPR to ECPR after a lag of 5 to 10 min [11]. However, when CCPR fails and ECPR is required, such a delay may result in worse patients outcomes compared to an immediate conversion to ECPR, since time to ECMO flow directly influences survival [12,13]. Ohbe et al. write in their recent analysis that “whether we should wait for the first 10 minutes of cardiac arrest without preparing for ECPR is questionable” [14]. These data show that there is currently no clear guidance as to how quickly to convert from CCPR to ECPR or which parameters should inform this decision.

Adding to this complexity is the fact that ECPR is a resource-intensive, side-effect-prone treatment modality, and instituting ECMO under CPR conditions can compromise the quality of conventional CPR efforts [15]. This issue is particularly important given that the quality of CPR has been shown to impact outcomes after resuscitation events [16]. Beyond the complexity of performing cannulation under CPR, ECMO itself is a high-risk treatment modality that is associated with significant morbidity and mortality [17]. Finding the balance between the lower complexity and lower treatment risk of conventional CPR and the higher complexity and higher treatment risk, but potentially better outcomes, of ECPR, currently remains driven by clinician assessment and opinion. Committing a patient early in the resuscitation process to ECPR may be beneficial, as this may shorten the time to ECMO flow. Conversely, persisting with CCPR may be equally beneficial, as ECMO is a high-risk, resource-intensive treatment modality [14,15]. We were unable to find literature that defines pre-CPR-event parameters that could guide clinicians on when to convert from CCPR to ECPR.

Given the uncertainties around the timing of ECPR and the potentially detrimental delays in deploying it, we conducted the present study aiming to improve our understanding of which patients may benefit from prompt conversion from CCPR to ECPR. The study’s primary aim was to establish pre-CPR-event parameters that could inform clinicians on whether CCPR would likely be successful or whether it may have a high likelihood of failing, thus making immediate activation of the ECPR process imperative. In addition, we sought to establish the outcomes of CCPR and ECPR events, respectively, and compare how they may differ between these two treatment modalities. Further, we aimed to establish how continuing CCPR or converting to ECPR in patients that had a CPR event lasting longer than 10 min influenced outcomes. Lastly, we analysed the time trajectory of mortality, whether it occurred early or late after CPR events, and whether mortality ceased to occur in patients that experienced a CPR event in the PICU.

## 2. Materials and Methods

We performed a retrospective analysis of paediatric in-hospital events requiring CPR that occurred in a tertiary mixed cardiac and medical Paediatric Intensive Care Unit (PICU). First, we assessed whether any pre-CPR-event variables were associated with the need to convert from CCPR to ECPR. Second, we analysed the survival outcomes for either event.

Patients were identified via an institutional database, where all CPR events in the PICU are recorded.

Inclusion criteria were: age less than 18 years, admission to PICU between 2016 and 2022, and occurrence of an event that required cardiopulmonary resuscitation (with cardiac compressions) in PICU. We used the definition of “event requiring CPR” or “CPR-event” rather than “cardiorespiratory arrest”, as the distinction between severe bradycardia versus asystolic arrest is not clearly defined, and more recent guidelines recommend commencing CPR when there are no signs of life rather than being guided by the specifics of a detected or diagnosed rhythm [18]. Patients were excluded if their CPR event occurred outside of the PICU, including out-of-hospital CPR events. We recorded the duration of the CPR event, from the commencement of CPR until the return of spontaneous circulation (ROSC). For ECPR cases, we only recorded the time to ECMO flow, independently of whether they had ROSC prior to the commencement of ECMO flows. ECPR was defined according to the Extracorporeal Life Support Organisation (ELSO), which stipulates that ECPR is defined as ECMO cannulation during active chest compressions as well as ECMO cannulation for patients that may have achieved ROSC up to 20 min, but where such ROSC is deemed unstable [19]. At our institution, the decision to provide ECPR is physician-led, though in principle all children with CPR events qualify for ECPR, unless they meet contraindications for ECMO support. Such contraindications include severe life limiting illnesses, pre-existing severe disease limiting cerebral functioning, severe coagulopathy, and technical inability to provide ECMO (such as severe thromboses). Our analysis was deemed a quality assurance activity by the local Ethics and Human Research Committee (HREC) and was therefore exempt from requiring HREC approval or patient consent for use of the data.

### 2.1. Data Extraction

All data were entered and stored in a Research Electronic Capture (REDCap) database hosted by the University of Queensland [20]. After CPR event data were entered, patient observation variables before and after the CPR event were extracted from the electronic medical record (EMR). Information on patient characteristics, background morbidity, and admission diagnoses were recorded. CPR event descriptors were also entered, such as the time of commencement of CPR, duration of CPR, number of doses of epinephrine administered during the event, time of return of spontaneous circulation (ROSC), whether the CPR event was preceded by a respiratory arrest, and the need for ECMO support. CPR event outcomes were measured on a patient level for PICU and hospital survival, PICU length-of-stay, and hospital length-of-stay. However, the outcome (survival vs. no survival) was also recorded for each CPR event. The pre-CPR-event observations were those recorded closest to the CPR event. For the laboratory measurements, a pragmatic time window of 4 h prior to the CPR event was chosen, during which laboratory data were recorded. For multiple results, the one closest to the CPR event was recorded. The following data were extracted: heart rate, mean arterial blood pressure, respiratory rate, blood gas parameters (pH and lactate), and serum creatinine concentrations. The pH was scaled by a factor of 10 prior to analysis, to aid in the interpretability of effect estimates.

### 2.2. Data Analysis

Data were cleaned and screened for outliers, missing values, and inconsistencies prior to analysis. Moderate proportions (28%) of missing observations were present for pre-CPR-event clinical measurements, with no missing values for all other reported variables.

Descriptive statistics were calculated for the CCPR and ECPR groups to summarise the participants’ baseline characteristics. Due to strongly skewed distributions, continuous variables were summarised using medians and interquartile ranges (IQRs), with frequencies and percentages reported for categorical variables.

Bivariate penalised logistic generalised estimating equation (GEE) models were constructed to assess the association between the patient characteristics, pre-CPR-event lab measurements, whether the CPR event was preceded by a respiratory arrest, and conversion from CCPR to ECPR. Penalised logistic GEEs, as described by Geroldinger et al., were applied to account for quasi-complete separation in the multivariable setting and the occurrence of repeated cardiac arrests within PICU admissions [21]. The dependence between repeated arrests violates the independence assumption of standard regression methods. GEE models, like logistic mixed-effects regression models, account for this dependence enabling accurate estimation of the confidence interval for covariate effects [22]. The former was chosen over the latter in this analysis, as population-level effect estimates were of interest. An exchangeable correlation structure was assumed between arrests in the penalised GEE model, selected based on the minimisation of the quasi-information likelihood criteria [23]. Cluster robust standard errors were calculated for effect estimates, and model specification was assessed through visual assessment of residual plots. All covariates evaluated in the bivariate setting, excluding admission weight, were included in a multivariable penalised logistic GEE model to assess their association with the outcome upon controlling for baseline characteristics and potential confounders. These covariates included age, sex, admission diagnosis, pre-CPR plasma lactate, pre-CPR pH, and preceding respiratory arrest. Weight was excluded from the multivariable model due to substantial collinearity with age and superior model fit with the inclusion of the latter.

Complete-case analyses were conducted using the bivariate and multivariable penalised logistic GEE models. Additionally, a sensitivity analysis was performed where missing pre-CPR-event measurement observations were imputed prior to model construction using multiple imputation by chained equations [24]. Linear mixed-effects regression was used for imputation, with all covariates in the multivariable model included as fixed effects and PICU admission identifiers included as random intercepts. One hundred imputed datasets were generated, with bivariate and multivariable models fit to the imputed datasets and estimated odds ratios and confidence intervals combined using Rubin’s rules [24]. Odds ratios (ORs) and 95% confidence intervals (95% CIs) for both the complete-case analysis and multiple imputation were reported for the bivariate and multivariable models. Kaplan–Meier analysis was conducted to assess differences in survival times following the respective CPR events between the ECPR and CCPR groups. Survival time was censored at the time of commencement of a subsequent CPR event, PICU discharge, or 90 days following the CPR event, with all-cause mortality as the event of interest.

As this is a convenience sample, the study is not powered for statistical comparisons and therefore *p*-values are not reported. Analyses were undertaken using the R programming language (version 4.4.0), the modified geefirthr package by Geroldinger et al., and the mice and survival packages [21,25,26].

## 3. Results

### 3.1. Demographics

A total of 201 CPR events occurred between January 2016 and December 2022 in 164 children in the PICU, over a total of 160 hospital admissions. Of these 201 CPR events, 45 (22.4%) led to ECMO support. The ECPR group versus the CCPR group consisted of a higher proportion of neonates (55.3% vs. 27.0%) and patients with cardiac surgical diseases, as compared with other diseases (55.3% vs. 32.5%; Table 1). In the CCPR group, six patients were ECMO supported electively, unrelated to an acute resuscitation event. These patients were included in the “CCPR” group only.

### 3.2. CPR Event Characteristics

CPR events occurred primarily outside of daylight hours (62.2%) and were predominantly asystolic and/or bradycardic episodes (57.2%). The median time to ROSC was 2 min (IQR: 1, 5) for the CCPR group. For the ECPR group, the median time to ECMO flow was 43 min (IQR: 30, 64). ROSC was achieved in 93.6% of CCPR events. Patients with CCPR received a median of 1 dose (IQR: 0, 2) of epinephrine versus 7 doses (IQR: 4, 11) that were given in ECPR patients (Table 2). Pre-CPR-event laboratory values were similar for creatinine, pH, and pCO2. The median serum lactate was 1.8 mmol/L (IQR: 1.0, 3.6) in the CCPR and 4.5 mmol/L (IQR: 1.6, 7.4) in the ECPR group. 35.3% of CCPR and 4.4% of ECPR events were preceded by a respiratory arrest. The pre-CPR-event mean arterial pressure and heart rate did not differ between the two groups (Table 2). 22 CCPR events lasted longer than 10 min and did not convert to ECPR. Reasons for not converting to ECPR included ROSC (*n* = 7), severe pulmonary hypertension with uncertain cerebral blood flow during the CPR event (*n* = 2), and uncertainty about long-term outcomes due to either cardiac disease that was deemed not to have surgical options, oncologic disease, or genetic syndromes (*n* = 13).

Of the 156 CCPR events, 22 (14.1%) lasted longer than 10 min. In contrast, 43 (95.6%) of the 45 ECPR events lasted longer than 10 min. Six (4.8%) patients in the CCPR group received elective ECMO support during their PICU stay; however, they did not receive ECPR and are represented in the CCPR group, not the ECPR group. Three patients in the ECPR group had a CPR event while on ECMO and re-establishing ECMO flow took less than 10 min.

### 3.3. Outcomes

The duration of mechanical ventilation was 6 days (median, IQR: 3, 16) in the CCPR versus 14 (IQR: 9, 30) days in the ECPR group. The median PICU length of stay was 13 (IQR: 6, 31) vs. 19 (IQR: 9, 34) days, and the median hospital lengths of stay were 37 (IQR: 14, 94) vs. 39 (IQR: 19, 65) days in the CCPR and ECPR groups, respectively. Eleven (7.1%) patients died within 2 hours of their CPR event in the CCPR group versus none in the ECPR group. The PICU mortality was 27.8% in the CCPR and 50% in the ECPR group, with the hospital mortality showing similar numbers. Four patients were transferred out of the PICU, resulting in a denominator of 160 patients for the hospital mortality.

A total of 54 patients died. The admission diagnosis of “Cardiac Surgical” was associated with the highest mortality. Of the 19 deaths in the ECPR group, 10 (52.6%) were admitted for cardiac surgical reasons. All ECPR events occurred after their surgical repair.

Of the 134 CCPR events that lasted less than or equal to 10 min, 21 (15.7%) led to death. Of the 22 CCPR events that lasted longer than 10 min, 15 (68.2%) resulted in death (Table 3).

### 3.4. Timing and Reason for Demise

**Figure 1:**
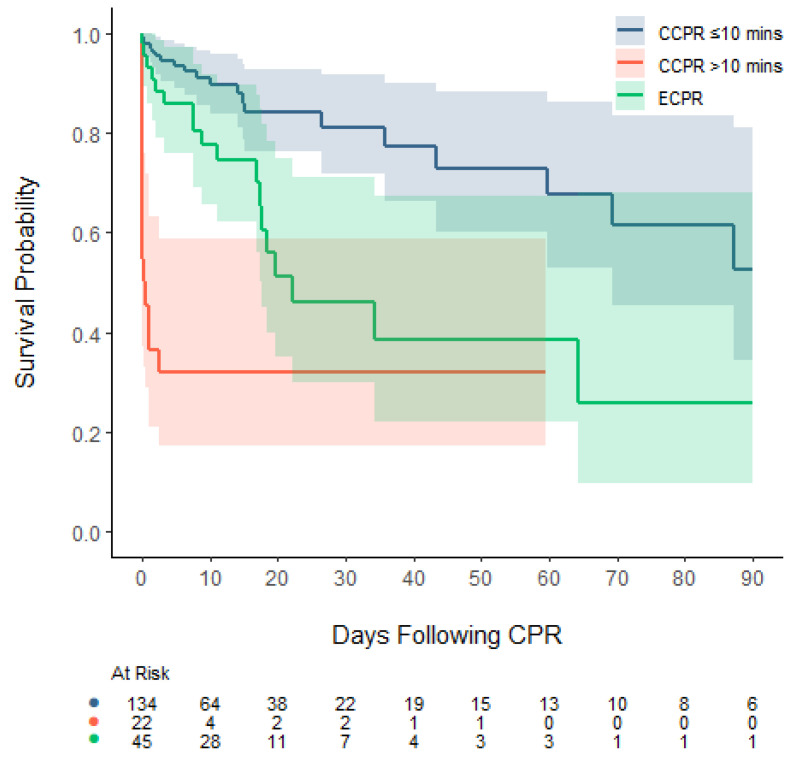
Kaplan–Meier plot (with 95% confidence intervals) of CPR event survival censored at 90 days of PICU admission, stratified by CCPR ≤ 10 min, CCPR > 10 min, and ECPR.

Deaths occurred at a median of 3 days (IQR: 0, 17) after the CPR event. After ECPR, deaths occurred at a median of 10 days (IQR: 3, 18), versus a median of one day (IQR: 0, 15) for patients after CCPR. After ECPR, deaths occurred in an initial period of up to 64 days, whereas after CCPR, deaths continued to occur up to 87 days post-arrest (Figure 1). Amongst CPR-events lasting longer than 10 min, deaths occurred at a median of 9 days (IQR: 2, 18) following ECPR and 0 days (IQR: 0, 0) following CCPR (Table 3).

The majority of deaths (74.1%) occurred due to elective withdrawal of life-sustaining treatments due to the severity of the overall illness. In the ECPR group, this amounted to 89.5% of deaths, whereas in the CCPR group, life-sustaining therapies were ceased in 65.7% (Table 3).

In nine events (4.5%), ROSC could not be achieved. In over 50% of cases, this was due to pulmonary hypertensive crises. None of these patients were ECPR supported.

In four patients, death occurred due to illnesses or events that were unrelated to the initial condition that necessitated CPR. Cardiac conditions were the most common cause of death among both the ECPR (57.9%) and CCPR (28.6%) groups.

### 3.5. Bivariate and Multivariable Analysis

In both the complete-case analysis and analysis following multiple imputation, serum lactate concentration was positively associated with a conversion from CCPR to ECPR in the bivariate and multivariable settings, while preceding respiratory arrest was negatively associated with conversion across these settings. An association was observed in the bivariate setting between admission diagnosis and conversion from CCPR to ECPR in both the complete case and multiple imputation analysis; however, it was not present in the multivariable setting. No associations were observed between conversion from CCPR to ECPR and age, sex, or pH across all analyses and settings (Table 4).

## 4. Discussion

Predicting which patients require escalation from CCPR to ECPR during a CPR event remains challenging, and data in the literature are lacking. Where CCPR and ECPR events are compared, comparisons focus on outcome differences between the two when used for resuscitation rather than factors that may predict a conversion from CCPR to ECPR [27,28,29]. To deepen our understanding of resuscitation events and the need for ECPR, we attempted to identify pre-CPR-event factors associated with the need for ECPR and those associated with the success of CCPR.

We found that cardiac surgical diagnosis and pre-CPR-event serum lactate levels were positively associated with conversion to ECPR. This demonstrates that the presence of these patient or laboratory findings should alert clinicians to the increased risk that a CCPR event may need escalation to ECPR. On the other hand, the occurrence of a respiratory arrest prior to the CPR event was negatively associated with conversion to ECPR, indicating that this group may be unlikely to require ECPR. The duration of CPR was highly predictive of conversion from CCPR to ECPR, though this is not a surprising finding, given that ECPR is instituted after CCPR is unsuccessful for a finite period of time.

While the benefits of ECPR for out-of-hospital cardiac arrests continue to be debated, some data suggest that for in-hospital cardiac arrest (IHCA) there may be a benefit when offering ECPR [7,27,28,30]. Most recently, Kobayashi demonstrated that ECPR improved survival when CPR continued for more than 10 min [9]. All these analyses follow the clinical reality where ECPR is usually decided on after 5 min of CPR and implemented after 30 to 40 min, mainly due to the complexity of the ECMO cannulation process [11]. Our study shows a similar finding, where mortality in CPR events that lasted more than 10 min was very high, should CCPR continue and ECPR not be employed. In fact, after 10 min of CPR, survival in the CCPR group was inferior to survival in the ECPR group—a finding that is similar to that of Kobayashi et al. [9]. The Kaplan–Meier graph shows how survival can be divided into three groups: patients who experienced CPR events shorter than 10 min, patients who received ECPR, and those who experienced CPR-events longer than 10 min who did not receive ECPR. Given this is a retrospective study, the comparison between CCPR and ECPR lasting greater than 10 min can result in a comparison of disparate groups, given the two cohorts may represent different disease states and illness severities. However, as outlined above, in the CCPR group, the majority of patients did not qualify for ECPR, due to considerations of their long-term rather than their short-term outcomes, and the trends we observed may therefore still provide insight into the value of ECPR versus CCPR in patients with prolonged CPR events. Our data suggest that using ECPR over CCPR after short CPR events may not be associated with higher survival rates, whereas for longer CPR events, the converse may be true, where ECPR may be associated with higher survival rates compared to CCPR. These findings raise questions about the threshold benefit of ECPR versus CCPR, similar to observations in ECMO provision, where survival is poorer in low-risk patients but higher in high-risk patients, albeit for sepsis and congenital diaphragmatic hernia patients rather than those experiencing undefined CPR events [31,32]. While different survival rates for different initial cardiac rhythms have been described, the distribution of the type of initial rhythms showed mostly bradycardic or asystolic events, and a low amount of PEA cases, a finding that differs from the published literature [33]. This is most likely due to the fact that CPR was commenced early, without attempts to define the initial cardiac rhythm. Therefore, PEA events may have been more common than described elsewhere in the literature [34].

Hospital survival rates were approximately 50% and 70% for patients who received ECPR and CCPR, respectively. The ECPR survival for this cohort was lower than our previously reported ECPR outcomes over a longer time period, likely due to variations in survival between years, and variations in illness severity of patients that were supported with ECMO [10]. The majority of deaths in the ECPR group occurred due to elective withdrawal of life-sustaining therapies. Mortality after ECPR primarily reflected the severity of the underlying condition rather than complications of ECMO. This demonstrates the initial effectiveness of ECPR and the uncertainty of long-term survival at the time of ECPR institution.

Our hospital survival rate of 70% after CCPR events compares favourably with the literature, where IHCA survival is reported at around 50%, most recently by Frazier et al. [4]. Our numbers may well be higher due to the fact that very short CPR events were included in our cohort. We included a Kaplan–Meier analysis in our report, given that the trajectory of survival is a finding that, to our knowledge, has not been reported before in the literature. While mortality after ECPR seems to occur slightly later than mortality after CCPR events, it plateaus after 64 days. In contrast, the CCPR mortality, while overall lower than that of the ECPR group, likely due to a lower disease severity pre-CPR event, continues to occur longer after the CPR event. Thus, IHCA events may be seen as a risk factor for ongoing mortality, possibly well into the future of a patient’s journey.

## 5. Limitations

The retrospective nature of our analysis limits the comparability of the ECPR and CCPR groups: some patients in the CCPR group who received prolonged resuscitation may not have been offered ECPR due to factors clinicians considered at the time of resuscitation, thereby limiting their comparability. Additionally, although sensitivity analyses comparing the results of complete-case analysis and multiple imputation indicated similar findings, residual bias may remain in the reported effect estimates depending on the mechanism by which missing data arose. Bias may also have been introduced through the omission of unmeasured confounding variables in the model. Our study was limited to patients within the PICU. Patients outside the PICU are not monitored as closely as those in the PICU, and had we included these patients in our study, there would have been a larger number of missing pre-CPR-event observations. This limitation to PICU patients, however, limits the generalisability of our findings. Clinician practice may vary regarding thresholds for ECPR over CCPR, which may have introduced bias into our results. However, the ECMO call-out system in our hospital involved multiple staff who together decide on ECMO eligibility, thereby reducing individual clinician practice variability. Lastly, our study is a single-centre study, involving institutional practices that may vary compared to other centres.

## 6. Conclusions

Predicting the need for ECPR at the outset of a CCPR event remains challenging. Pre-CPR-event serum lactate concentrations are positively associated with the need to convert from CCPR to ECPR and may, therefore, serve as markers indicating a higher likelihood of requiring ECMO support. Conversely, respiratory arrest prior to CPR events is negatively associated with conversion to ECPR and may indicate a lower likelihood of needing ECMO support. Any CCPR event should alert clinicians that, even if the patient recovers from the event, their mortality risk remains high, even at a time well after the CPR event has been resolved. We suggest that further research is needed to identify the factors that may be associated with conversion from CCPR to ECPR and those that contribute to the success of CCPR, in order to avoid the unnecessary use of ECMO, given its high resource intensity and cost.

## Figures and Tables

**Table 1:** Patient characteristics (CCPR: conventional cardiopulmonary resuscitation; ECPR: ECMO-assisted cardiopulmonary resuscitation; PICU: Paediatric Intensive Care Unit; ECMO: extracorporeal membrane oxygenation; IQR: interquartile range).

Characteristic	Total *N* = 164	CCPR *N* = 126	ECPR *N* = 38
Age, *n* (%) Neonate (<28 days) 28 days to 1 year 1 year to 5 years >5 years	55 (33.5%) 54 (32.9%) 25 (15.2%) 30 (18.3%)	34 (27.0%) 46 (36.5%) 22 (17.5%) 24 (19.0%)	21 (55.3%) 8 (21.1%) 3 (7.9%) 6 (15.8%)
Weight (kg), median (IQR)	4.7 (3.3, 13.1)	5.1 (3.4, 14.3)	3.8 (3.0, 8.6)
Sex, *n* (%) Male Female	85 (51.8%) 79 (48.2%)	68 (54.0%) 58 (46.0%)	17 (44.7%) 21 (55.3%)
Primary PICU diagnosis, *n* (%) Cardiac Surgical Other	62 (37.8%) 102 (62.2%)	41 (32.5%) 85 (67.5%)	21 (55.3%) 17 (44.7%)

**Table 2:** CPR event characteristics (CCPR: conventional cardiopulmonary resuscitation; ECPR: ECMO-assisted cardiopulmonary resuscitation; PEA: pulseless electrical activity; VF: ventricular fibrillation; VT: ventricular tachycardia; ROSC: return of spontaneous circulation; ECMO: extracorporeal membrane oxygenation; MAP: mean arterial pressure; HR: heart rate; IQR: interquartile range).

Characteristic	Variable	Total *N* = 201	CCPR *N* = 156	ECPR *N* = 45
Time of day	7 a.m.−5 p.m., *n* (%)	76 (37.8%)	58 (37.2%)	18 (40.0%)
	5 p.m.−7 a.m. (after hours), *n* (%)	125 (62.2%)	98 (62.8%)	27 (60.0%)
Rhythm	Asystolic/Bradycardic CPR event, *n* (%) PEA, *n* (%) VF/VT, *n* (%) Respiratory arrest followed by cardiac arrest, *n* (%) Unknown, *n* (%)	115 (57.2%) 12 (6.0%) 16 (8.0%) 57 (28.4%) 1 (0.5%)	82 (52.6%) 6 (3.9%) 12 (7.7%) 55 (35.3%) 1 (0.6%)	33 (73.3%) 6 (13.3%) 4 (8.9%) 2 (4.4%) 0 (-)
CPR event details	Time to ROSC (min), median (IQR)	3 (2, 20)	2 (1, 5)	37 (21, 60)
	Elective ECMO support received, *n* (%)	44 (26.8%) *(N* = 164)	6 (4.8%) (*N* = 126)	38 (100%) (*N* = 38)
	Any ROSC, *n* (%)	164 (81.6%)	146 (93.6%)	18 (40.0%)
	Time to ECMO flow (min), median (IQR) All ROSC Achieved ROSC Not Achieved	- - -	- - -	43 (30, 64) 43 (28, 75) (*N =* 18) 43 (30, 61) (*N* = 27)
	CPR duration > 10 min, *n* (%)	65 (32.3%)	22 (14.1%)	43 (95.6%)
	CPR preceded by respiratory arrest, *n* (%)	57 (28.4%)	55 (35.3%)	2 (4.4%)
	Number of adrenaline doses, *n* (%)	1 (1, 5)	1 (0, 2)	7 (4, 11)
Pre-ECPR laboratory measurements (last within 180 to 5 min pre-CCPR or ECPR)	Baseline creatinine (µmol/L), median (IQR)	52.0 (30.0, 70.5)(*N* = 55)	45.0 (30.0, 72.5) (*N* = 35)	56.5 (33.8, 66.2) (*N* = 20)
	pH, median (IQR)	7.3 (7.2, 7.4) (*N* = 146)	7.3 (7.2, 7.4) (*N* = 107)	7.3 (7.2, 7.3) (*N* = 39)
	pCO2 (mmHg), median (IQR)	44.0 (36.0, 54.0) (*N* = 147)	46.0 (39.0, 57.2) (*N* = 108)	38.0 (32.0, 45.0) (*N* = 39)
	Lactate (mmol/L), median (IQR)	2.3 (1.1, 4.6) (*N* = 144)	1.8 (1.0, 3.6) (*N* = 106)	4.5 (1.6, 7.4) (*N* = 38)
	MAP (mmHg), median (IQR)	57 (46, 68) (*N* = 190)	59 (48, 70) (*N* = 148)	49 (38, 57) (*N* = 42)
	HR (beats per minute), median (IQR)	138 (113, 155) (*N* = 196)	135 (109, 152) (*N* = 154)	147 (128, 160) (*N* = 42)

**Table 3:** Patient and CPR event outcomes (CCPR: conventional cardiopulmonary resuscitation; ECPR: ECMO-assisted cardiopulmonary resuscitation; ECMO: extracorporeal membrane oxygenation; PICU: Paediatric Intensive Care Unit; LOS: length of stay; ROSC: return of spontaneous circulation; IQR: interquartile range).

Characteristics		Total	CCPR	ECPR
Hospital stay	Duration of ECMO (days), median (IQR)	5 (3, 10) (*N* = 51)	4 (4, 6) (*N* = 6)	5 (3, 11) (*N* = 45)
	Duration of mechanical ventilation (days), median (IQR)	8 (3, 19) (*N* = 164)	6 (3, 16) (*N* = 126)	14 (9, 30) (*N* = 38)
	PICU LOS (days), median (IQR)	16 (7, 32) (*N* = 164)	13 (6, 31) (*N* = 126)	19 (9, 34) (*N* = 38)
	Hospital LOS (days), median (IQR)	39 (15, 85) (*N* = 160)	37 (14, 94) (*N* = 122)	39 (19, 65) (*N* = 38)
Mortality	Mortality within 2 h after ROSC, *n* (%)	11 (5.5%) (*N* = 201)	11 (7.1%) (*N* = 156)	0 (-) (*N* = 45)
	Mortality by CPR event length, *n* (%) CPR event lasting > 10 min CPR event lasting ≤ 10 min	32 (49.2%) (*N* = 65) 22 (16.2%) (*N* = 136)	15 (68.2%) (*N* = 22) 21 (15.7%) (*N* = 134)	17 (39.5%) (*N* = 43) 1 (50.0%) (*N* = 2)
	PICU mortality, *n* (%)	54 (32.9%) (*N* = 164)	35 (27.8%) (*N* = 126)	19 (50.0%) (*N* = 38)
	Hospital mortality, *n* (%)	54 (33.8%) (*N* = 160)	35 (28.7%) (*N* = 122)	19 (50.0%) (*N* = 38)
	Mortality by primary PICU diagnosis, *n* (%) Cardiac Surgical Other	17 (31.5%) 37 (68.5%) (*N* = 54)	7 (20.0%) 28 (80.0%) (*N* = 35)	10 (52.6%) 9 (47.4%) (*N* = 19)
	Time to death (days), median (IQR) All CPR event lasting > 10 min	3 (0, 17) (*N* = 54) 1 (0, 9) (*N* = 32)	1 (0, 15) (*N* = 35) 0 (-) (*N* = 15)	10 (3, 18) (*N* = 19) 9 (2, 18) (*N* = 17)
Cause of death	Cause of death main classification, *n* (%) Unable to achieve ROSC Elective withdrawal due to underlying illness severity Mechanical difficulty during ECPR Death unrelated to arrest event	9 (16.7%) 40 (74.1%) 1 (1.9%) 4 (7.4%) (*N* = 54)	9 (25.7%) 23 (65.7%) 0 (-) 3 (8.6%) (*N* = 35)	0 (-) 17 (89.5%) 1 (5.3%) 1 (5.3%) (*N* = 19)
	Cause of death subclassification, *n* (%) Cardiac Neurology Oncology Respiratory Sepsis Other	21 (38.9%) 9 (16.7%) 4 (7.4%) 8 (14.8%) 5 (9.3%) 7 (13.0%) (*N* = 54)	10 (28.6%) 7 (20.0%) 3 (8.6%) 6 (17.1%) 3 (8.6%) 6 (17.1%) (*N* = 35)	11 (57.9%) 2 (10.5%) 1 (5.3%) 2 (10.5%) 2 (10.5%) 1 (5.3%) (*N* = 19)

**Table 4:** Bivariate and multivariable complete-case analysis and multiple imputation analysis of need for conversion from CCPR to ECPR (OR: odds ratio; CI: confidence interval; aOR: adjusted odds ratio; CPR: cardiopulmonary resuscitation).

	Bivariate Complete Case *N* = 201	Bivariate Multiple Imputation *N* = 201	Multivariable Complete Case *N* = 144	Multivariable Multiple Imputation *N* = 201
Variable	OR (95% CI)	OR (95% CI)	aOR (95% CI) ^a^	aOR (95% CI) ^a^
Age (years)	0.98 (0.89, 1.08)	0.98 (0.89, 1.08)	0.98 (0.88, 1.09)	1.01 (0.91, 1.12)
Weight (kg)	0.99 (0.96, 1.02)	0.99 (0.96, 1.02)	-	-
Sex Female (reference) Male	- 0.81 (0.38, 1.73)	- 0.81 (0.38, 1.73)	- 0.73 (0.29, 1.82)	- 0.84 (0.37, 1.91)
Diagnosis Other (reference) Cardiac Surgical	- 2.61 (1.21, 5.60)	- 2.61 (1.21, 5.60)	- 1.70 (0.68, 4.23)	- 2.26 (0.99, 5.17)
Lactate (pre-CPR event)	1.13 (1.04, 1.24) (*N =* 144)	1.15 (1.06, 1.24)	1.14 (1.02, 1.27)	1.15 (1.04, 1.28)
pH (pre-CPR event)	0.86 (0.69, 1.08) (*N =* 146)	0.88 (0.70, 1.11)	1.16 (0.79, 1.70)	1.10 (0.78, 1.56)
Preceeding respiratory arrest No (reference) Yes	- 0.12 (0.04, 0.37)	- 0.12 (0.04, 0.37)	- 0.20 (0.06, 0.65)	- 0.13 (0.04, 0.41)

^a^ Adjusted for all variables in bivariate analysis excluding weight.

## Data Availability

The data presented in this study are available on request from the corresponding author after data-sharing agreements have been established.

## Abbreviations

The following abbreviations are used in this manuscript:

CPR	Cardiopulmonary resuscitation
CCPR	Conventional cardiopulmonary resuscitation
ECMO	Extracorporeal membrane oxygenation
ECPR	ECMO-assisted CPR
HREC	Human Research and Ethics Committee
IQR	Interquartile range
OT	Operating theatre
PICU	Paediatric Intensive Care Unit
ROSC	Return of spontaneous circulation
VA	Veno-arterial
VV	Veno-venous
